# Incidence of acute-onset endophthalmitis after separate bilateral cataract surgeries less than 5 days apart

**DOI:** 10.1186/s12886-019-1028-y

**Published:** 2019-01-25

**Authors:** Ying Chen, Yu Zhang, Xiaodan Li, Hong Yan

**Affiliations:** 1 Department of Ophthalmology, The First Affiliated Hospital of Chongqing Medical University, Chongqing Key Laboratory of Ophthalmology, Chongqing Eye Institute, Chongqing, 400016 China; 2 Department of Ophthalmology, Chongqing Traditional Chinese Medicine Hospital, Chongqing, 400021 China; 3 Department of Ophthalmology, Xi’an No. 4 Hospital, Shaanxi Eye Hospital, Affiliated Guangren Hospital, School of Medicine, Xi’an Jiaotong University, Xi’an, 710004 Shaanxi Province China

**Keywords:** Endophthalmitis, Bilateral cataract surgery

## Abstract

**Purpose:**

To assess the incidence of acute-onset endophthalmitis after separate bilateral cataract surgeries less than 5 days apart (SBCS5).

**Methods:**

The medical records of all patients who underwent SBCS5 at a single medical center between October 10, 2012 and July 31, 2017 were retrospectively reviewed.

**Results:**

The medical records for treatment of 5374 eyes of 2687 patients were examined. The mean interval between the first and second surgeries was 3 days. No case of bilateral simultaneous endophthalmitis was observed. Unilateral endophthalmitis developed in five eyes of five patients. Thus, the incidence of endophthalmitis after SBCS5 was 0.093%. All cases of endophthalmitis occurred in the first operated eye. SBCS5 was 15% less expensive than unilateral cataract surgery.

**Conclusion:**

The incidence of endophthalmitis after SBCS5 was acceptably low with topical but not intracamaral antibiotic prophylaxis. SBCS5 was also less expensive than unilateral cataract surgery.

## Background

Cataract surgery has advanced such that successful outcomes are achieved with minimal surgical complications. It is well established that patients with bilateral cataracts who undergo surgery in both eyes have greater improvement in visual function than those who undergo surgery in only one eye. [1, 2] Traditionally, only one eye is operated on at a time, and the timing of cataract surgery on the second eye has been the subject of research. [2, 3]

Over the past two decades, immediately sequential bilateral cataract surgery (ISBCS) was introduced [4, 5] and shown to be cost-effective compared with delayed sequential bilateral cataract surgery (DSBCS). Moreover, ISBCS provides rapid visual rehabilitation with normal stereopsis and binocular functionality and increased convenience for the patient with fewer clinical evaluations and hospital visits. [6, 7] However, ISBCS is not routinely performed due to concern over serious postoperative bilateral sight-threatening complications such as endophthalmitis. [8–10] Although rare, endophthalmitis is a disastrous complication that poses a significant public health issue, considering the millions of people who undergo cataract surgery every year.

‘Separate bilateral cataract surgeries several days apart’ (SBCS) with postoperative clinical observation can avoid some of the complications of ISBCS and still be as cost-effective and convenient for the patient as ISBCS. If a complication is noted in the first surgical eye, surgery on the second eye can be canceled or delayed. The vast majority of patients undergoing cataract surgery are reluctant to have the second eye operated on until they are sure of visual improvement in the first eye. Owing to the social, clinical, and economic advantages, SBCS is permitted by health insurance in some areas of China and has recently become a routine surgical practice in some ophthalmic centers. One potential disadvantage of SBCS is that acute endophthalmitis may present within 6 weeks after cataract surgery, and thus, that the second eye has been operated on before endophthalmitis in the first eye is detected. This raises concern over whether SBCS increases the incidence of endophthalmitis, and because of this, in some areas of China, the cost of SBCS is not reimbursed. It is imperative to know the incidence of endophthalmitis after SBCS within a short interval before SBCS is recommended. SBCS is routinely performed in our department with an interval between surgeries of less than 5 days to limit hospitalization costs. We therefore retrospectively reviewed the medical records of all patients who were treated with bilateral phacoemulsification and posterior chamber intraocular lens implantation less than 5 days apart (SBCS5) to determine the incidence of acute-onset endophthalmitis in either eye.

## Methods

The records of all patients who underwent SBCS5 in the Department of Ophthalmology, the First Affiliated Hospital of Chongqing Medical University, from October 10, 2012 to July 31, 2017 (*n* = 5374 eyes) were reviewed retrospectively. The patients’ age, gender, residence address, intraocular lens material, main incision type, preoperative and postoperative vision, and whether or not vitreous loss occurred were noted.

Patients who underwent SBCS5 were admitted to our hospital 1 day before or on the day of the first eye surgery. The eye with lower acuity was operated on first. Surgery on the second eye was performed by the same surgeon on the first to fifth postoperative day, and most often on the second postoperative day. The second eye surgery was canceled if a complication occurred in the first eye, such as communication with the vitreous, significant corneal edema, or refraction problems. Topical anesthesia was achieved with 0.4% oxybuprocaine hydrochloride. The lid margin and conjunctival sac were preoperatively sterilized with 1% povidone–iodine solution to disinfect, and the facial skin was treated with iodophor. The operative site was covered with autoclave sterilized drapes. A corneal incision was made, continuous curvilinear capsulorhexis performed, and the lens removed by phacoemulsification. A hydrophobic or hydrophilic acrylic intraocular lens was implanted in the bag.

All patients were instructed to use 0.3% levofloxacin from the day of admission to 2 weeks postoperatively. In addition, 0.1% pranoprofen and 1% prednisolone acetate were prescribed for 1 month and 3 weeks after surgery, respectively. Patients were permitted to leave the hospital 1 day after surgery if their eyes were considered normal upon slit-lamp examination. All patients visited their surgeon at the hospital at least twice within 1 month after surgery and were followed up at the hospital or by telephone at least once 2–3 months after surgery. The clinical manifestations, treatment strategies, and microbiology records of the patients diagnosed with endophthalmitis were reviewed. The frequency of endophthalmitis was calculated as the number of eyes affected by endophthalmitis divided by the total number of eyes operated on. The health-care costs of the SBCS5 were calculated.

Statistical analyses were performed using SPSS software. The Fisher exact test was used for comparison of endophthalmitis rates between the first and second operated eyes. A value of *P* < 0.05 was considered significant.

## Results

**Fig. 1:**
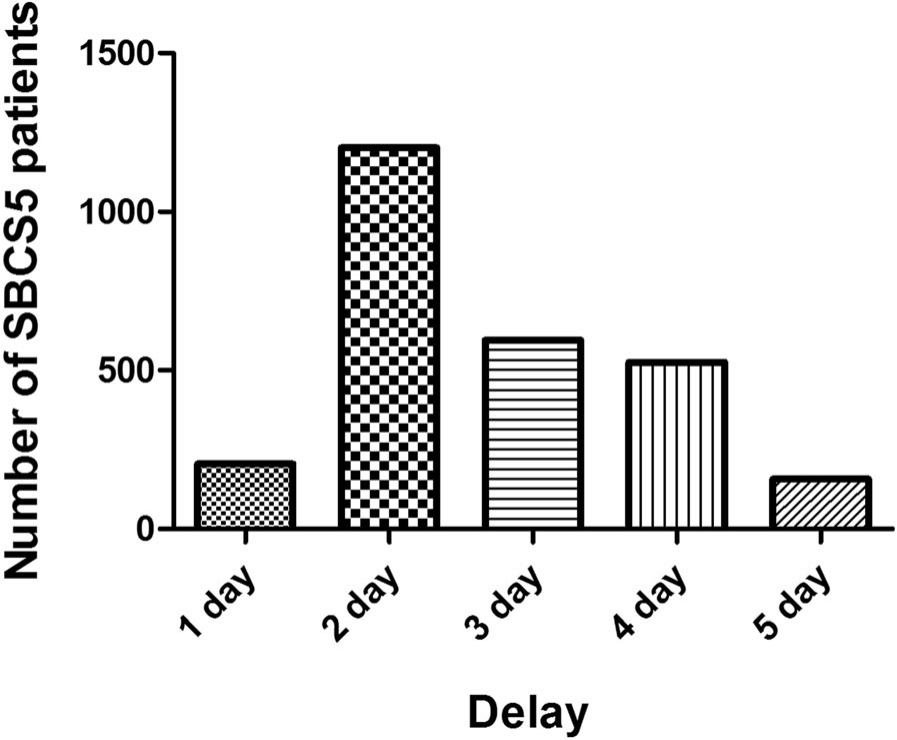
Distribution of time interval between the surgery on the first eye and the surgery on the second eye in SBCS5 patients

The medical records for 5374 eyes of 2687 patients who underwent SBCS5 were reviewed retrospectively. The clinical characteristics of the patients included in this study are described in Table 1. The mean time interval between the first and second eye surgeries was 3 days (Fig. 1). The metrics of preoperative and postoperative vision of the SBCS5 cohort are listed in Table 2. We did not exclude patients with retinopathy or optic neuropathy from the present study, and thus, the preoperative and postoperative vision results were affected by both cataract and posterior ocular diseases.

**Table 1:** Clinical characteristics of patients included in this study

Age (years)	72 ± 12 year
Gender	
Male	956 (36%)
Female	1731 (64%)
Diabetes	556 (20%)
Location	
Rural	846 (31%)
Urban	1841 (69%)
The order of operated eye	
Right/Left	1416 (53%)
Left/Right	1271 (47%)
Incision type	
Limbus	2212 (82%)
Clear corneal	475 (18%)
Vitreous loss	128 (2.4%)
Intraocular lens type	
Hydrophobic	890 (17%)
Hydrophilic	4484 (83%)

**Table 2:** Preoperative and postoperative vision of SBCS5 patients

Number				
	LogMAR vision (No.)	Counting finger	Hand move	Light perception
Preoperative	0.62 ± 0.35 (4359)	711	229	75
Postoperative	0.32 ± 0.32 (5224)	95	49	6

The health-care costs for SBCS5 per patient were 7147 RMB (¥) or 3574 RMB (¥) per eye. We randomly selected 1000 patients who underwent unilateral cataract surgery contemporaneously and calculated the health-care costs of their surgery to be 4215 RMB (¥), which was 18% more costly than SBCS5.

Endophthalmitis developed in five eyes of five patients for an incidence of 0.093%. No case of bilateral simultaneous endophthalmitis occurred. The mean age of the five patients with endophthalmitis was 64 years (range, 50–78 years). All five eyes with endophthalmitis (two right eyes and three left eyes) were eyes that had been operated on first. The difference in endophthalmitis rates between the eyes of the first and the second surgeries fell short of being statistically significant (*P* = 0.062). The clinical characteristics of the five patients with endophthalmitis are listed in Table 3. A clear corneal incision was made in two cases, for a rate of 0.21% in endophthalmitis cases and a rate of 0.068% in cases in which a limbus incision also was made. Three of the five patients lived in rural areas. The endophthalmitis rates of rural and urban residents were 0.35 and 0.11%, respectively.

**Table 3:** Clinical characteristics of patients with acute-onset postoperative endophthalmitis

Patient (Age range, Gender)	Diabetes	Location	Incision type	Intraocular lens type	Vitreous loss
1 (70–79, F)	Y	Rural	Clear corneal	Hydrophilic	No
2 (60–69, F)	N	Urban	Limbus	Hydrophilic	No
3 (50–59, F)	N	Urban	Limbus	Hydrophilic	No
4 (50–59, M)	N	Rural	Clear corneal	Hydrophilic	No
5 (50–59, F)	N	Rural	Limbus	Hydrophilic	No

At the time of presentation with endophthalmitis, all endophthalmitis cases had decreased vision after cataract surgery, four cases had increased redness, and one patient complained of significant pain. Hypopyon was observed in two of the five eyes. Intravitreal medications administered at the time of endophthalmitis diagnosis included vancomycin, ceftazidime, and dexamethasone. Of the five patients with endophthalmitis, two were initially treated with a pars plana vitrectomy. Table 4 shows the visual acuity, treatments, culture results, and outcomes for patients with acute-onset postoperative endophthalmitis.

**Table 4:** Visual Acuity, treatment, culture result and outcomes for Patients with acute-onset postoperative endophthalmitis

Patient	Time to diagnosis (d)	Delayed time of the second eye (d)	Visual acuity (logMAR) at Diagnosis	Management (Intravitreal Medications)	culture result	Final visual acuity (logMAR)
1	5	4	0.3	PPV/IM (V, C, D)	Negative	0.6
2	31	2	1	PPV/IM (V, C, D)	Negative	1
3	19	3	0.92	IM (V, C, D)	Negative	0.3
4	30	3	LP	PPV/IM (V, C, D)	Methicillin resistant coagulase-negative staphylococci	HM
5	3	2	CF	IM (V, C, D)	Staphylococcus epidermidis	0.3

*C* ceftazidime, *CF* counting finger, *D* dexamethasone, *HM* hand motion, *IM* intravitreal medications, *LP* light perception, *PPV* pars plana vitrectomy, *V* vancomycin

## Discussion

In our study, among all patients who underwent SBCS5, no case of bilateral simultaneous endophthalmitis was detected, which is consistent with the results of a retrospective study of 95,606 patients who underwent ISBCS without breach of an aseptic protocol. [11] Cases of bilateral simultaneous endophthalmitis have been reported to occur sporadically with ISBCS when the guidelines for strict separation of the two surgical procedures were not followed. [8, 9] The surgical procedure for SBCS5 specifying separate povidone iodine prepping, draping, instrumentation, different irrigating solutions, ophthalmic viscosurgical devices and medications likely contributed to the absence of any cases of bilateral simultaneous endophthalmitis occurring with SBCS5.

In our study, the incidence of endophthalmitis in the SBCS5 cohort was 0.093%, which is within the range of 0.03–0.3% reported in many large studies in and outside of China. [11–19] The cumulative incidence of endophthalmitis for eight eye centers of tertiary care hospitals (cataract surgery volume ≥ 150 cases per month) in China after cataract surgery was 0.033%, which was slightly lower than the rate observed in our study. [19] An approximately five-fold reduction in intracameral cefuroxime use was observed by the European Society of Cataract & Refractive Surgeons in a prospective study. [20] In our study, we used a topical rather than intracamaral antibiotic for anti-infective prophylaxis. A possible explanation for the difference in endophthalmitis rates after cataract surgery is the use of a different method of intraoperative anti-infective prophylaxis and the application of topical antibiotics in our eye center compared with several centers in the aforementioned study. An intracameral vancomycin injection (1 mg in 0.1 mL of normal saline) was used in one center, addition of antibiotics in irrigating solutions (tobramycin 16 mg/l or vancomycin 100 μg/ml in three centers, and subconjunctival antibiotics tobramycin, 8 or 4 mg/0.1 mL) in two centers. [19] In our study, we only used topical preoperative and postoperative antibiotic prophylaxis. The estimated endophthalmitis rates in the current study are based on the assumption that all patients who underwent surgery would return to our center in case of any complications. The patients were instructed to contact or visit their surgeon if any abnormal symptoms such as blurred vision, redness, or pain occurred in postoperative eyes and to follow up at the hospital or by telephone from 1 to 3 months after surgery. Therefore, it is unlikely that this study missed any patients who developed endophthalmitis and moved or sought care at another institution. One should note that it was possible that a case of super acute endophthalmitis presenting before the second eye surgery was not included in the calculation of endophthalmitis rates, because the second eye surgery would have been canceled, which could bias our data.

Of the five endophthalmitis cases observed, two were diagnosed by microorganism culture, and the others were diagnosed based on symptoms and signs with no positive proof of infection. The culture-positive rates in our study were lower than those in previous studies. [21, 22] Aqueous not vitreous samples were used for culture in two of our negative cases, which may have led to the negative culture results. [23, 24] Methicillin-resistant, coagulase-negative *staphylococci* (CoNS) and *Staphylococcus epidermidis* were isolated from two of our cases with endophthalmitis. Methicillin-resistant CoNS and *staphylococcus* are the most commonly isolated microorganisms from intraocular fluid and eyelid margin and conjunctival samples from patients with endophthalmitis after cataract surgery. [25, 26] The unexpected finding in the present study is that endophthalmitis was more common in the first operated eye than in the second operated eye despite the absence of statistical significance. A possible reason for this is that the second operated eye received topical antibiotics prophylaxis for a longer period of time than did the first operated eye. We administered topical antibiotics bilaterally so that the second eye received topical antibiotic prophylaxis for 1–5 days more than the first eye before cataract surgery, which possibly could have removed bacteria from the conjunctival sac more effictively.

One disadvantage of SBCS5 compared with traditional bilateral cataract surgeries separated by a long period of time is that because the refraction of the first surgical eye is unstable due to corneal edema or a change in the position of the intraocular lens after surgery, the refraction of the second surgical eye cannot be adjusted based on the refraction of the first-eye undergoing surgery, especially for myopic or hyperopic eyes.

The limitations of the present study include the review of records from only a single medical center, the small sample size, and the retrospective nature of the study. Further studies should be multi-centered with a prospective design.

## Conclusion

This is the first report of the frequency and cost of postoperative endophthalmitis after SBCS5, and based on the results, SBCS5 was found to be acceptable for use in a tertiary hospital in a developing country.

## Abbreviations

DSBCS: Delayed sequential bilateral cataract surgery
ISBCS: Immediately sequential bilateral cataract surgery
SBCS5: Separate bilateral cataract surgeries less than 5 days apart
